# 7-Benzenesulfonamido-3-methyl-8-oxo-5-thia-1-azabicyclo[4.2.0]octa-2-ene-2-carboxylic acid monohydrate

**DOI:** 10.1107/S1600536809051769

**Published:** 2009-12-09

**Authors:** Shahzad Sharif, Mehmet Akkurt, Islam Ullah Khan, Manan Ayub Salariya, Sarfraz Ahmad

**Affiliations:** a Materials Chemistry Laboratory, Department of Chemistry, Government College University, Lahore 54000, Pakistan; b Department of Physics, Faculty of Arts and Sciences, Erciyes University, 38039 Kayseri, Turkey; c Pharmagen Ltd, Lahore 54000, Pakistan; d Pharmagen Ltd., Lahore 54000, Pakistan

## Abstract

In the title compound, C_14_H_14_N_2_O_5_S_2_·H_2_O, the six-membered ring fused to the β-lactam unit has a twisted conformation. Weak intra­molecular N—H⋯S and C—H⋯O inter­actions occur. Inter­molecular C—H⋯S, N—H⋯O, C—H⋯O and O—H⋯O hydrogen-bonding inter­actions stabilize the crystal structure, forming a three-dimensional network. Weak C—H⋯π inter­actions are also present.

## Related literature

For the production of 7-amino-deacetoxy­cephalosporanic acid-like components by direct fermentation, see: Schroen *et al.* (2000 ▶). For 7-benzene­sulfonamido-3-ethenyl-8-oxo-5-thia-1-aza­bicyclo­[4.2.0]oct-2-ene-2-carboxylic acid methanol solvate, see: Mariam *et al.* (2009 ▶). For structures with the β-lactam unit, see: Akkurt *et al.* (2008*a* ▶,*b* ▶,*c* ▶); Baktır *et al.* (2009 ▶); Pınar *et al.* (2006 ▶); Yalçın *et al.* (2009 ▶); Çelik *et al.* (2009*a* ▶,*b* ▶). For puckering parameters, see: Cremer & Pople (1975 ▶). 
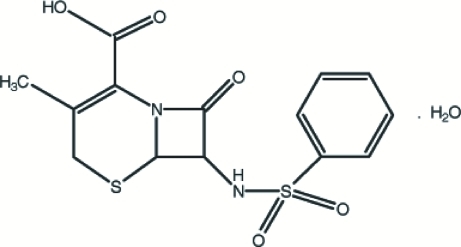


## Experimental

### 

#### Crystal data

C_14_H_14_N_2_O_5_S_2_·H_2_O
*M* *_r_* = 372.43
Orthorhombic, 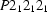

*a* = 5.9535 (7) Å
*b* = 15.8248 (19) Å
*c* = 18.411 (2) Å
*V* = 1734.6 (3) Å^3^
*Z* = 4
Mo *K*α radiation
μ = 0.34 mm^−1^
*T* = 296 K
0.20 × 0.20 × 0.20 mm

#### Data collection

Bruker Kappa APEXII CCD area-detector diffractometer
10847 measured reflections
3572 independent reflections
1890 reflections with *I* > 2σ(*I*)
*R*_int_ = 0.064

#### Refinement

*R*[*F* ^2^ > 2σ(*F* ^2^)] = 0.055
*wR*(*F* ^2^) = 0.109
*S* = 0.97
3572 reflections
226 parameters
3 restraints
H atoms treated by a mixture of independent and constrained refinement
Δρ_max_ = 0.20 e Å^−3^
Δρ_min_ = −0.25 e Å^−3^
Absolute structure: Flack (1983 ▶), 1425 Friedel pairs
Flack parameter: −0.07 (11)

### 

Data collection: *APEX2* (Bruker, 2007 ▶); cell refinement: *SAINT* (Bruker, 2007 ▶); data reduction: *SAINT*; program(s) used to solve structure: *SIR97* (Altomare *et al.*, 1999 ▶); program(s) used to refine structure: *SHELXL97* (Sheldrick, 2008 ▶); molecular graphics: *ORTEP-3 for Windows* (Farrugia, 1997 ▶); software used to prepare material for publication: *WinGX* (Farrugia, 1999 ▶) and *PLATON* (Spek, 2009 ▶).

## Supplementary Material

Crystal structure: contains datablocks global, I. DOI: 10.1107/S1600536809051769/vm2014sup1.cif

Structure factors: contains datablocks I. DOI: 10.1107/S1600536809051769/vm2014Isup2.hkl

Additional supplementary materials:  crystallographic information; 3D view; checkCIF report

## Figures and Tables

**Table 1:** Hydrogen-bond geometry (Å, °)

*D*—H⋯*A*	*D*—H	H⋯*A*	*D*⋯*A*	*D*—H⋯*A*
O1—H1⋯O*W*1^i^	0.82	1.86	2.663 (6)	166
O*W*1—H*W*1⋯O5^ii^	0.83 (4)	2.14 (5)	2.799 (6)	136 (5)
N2—H2⋯S1	0.86	2.82	3.136 (3)	103
N2—H2⋯O2^iii^	0.86	2.30	2.846 (4)	122
O*W*1—H*W*2⋯O3	0.84 (5)	2.54 (5)	3.135 (6)	129 (5)
C3—H3*A*⋯O1	0.96	2.22	2.902 (6)	127
C6—H6⋯S1^iv^	0.98	2.80	3.756 (4)	165
C8—H8⋯O4^i^	0.98	2.30	3.100 (5)	138
C11—H11⋯O3^v^	0.93	2.51	3.192 (6)	130
C3—H3*B*⋯*Cg*3^ii^	0.96	2.72	3.640 (5)	161

Symmetry codes: (i) 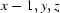; (ii) 
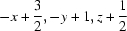
; (iii) 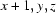; (iv) 
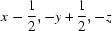
; (v) 
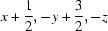
. *Cg*3 is the centroid of the C9–C14 ring.

**Fig. 1.**
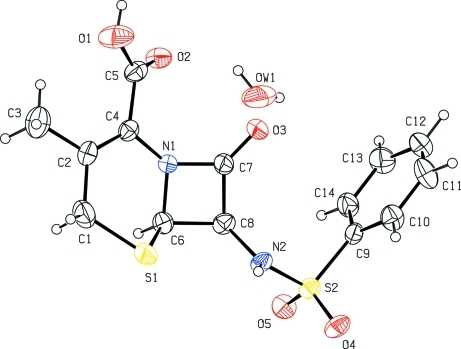
The title molecule with the atom numbering scheme. Displacement ellipsoids for non-H atoms are drawn at the 30% probability level.

**Fig. 2.**
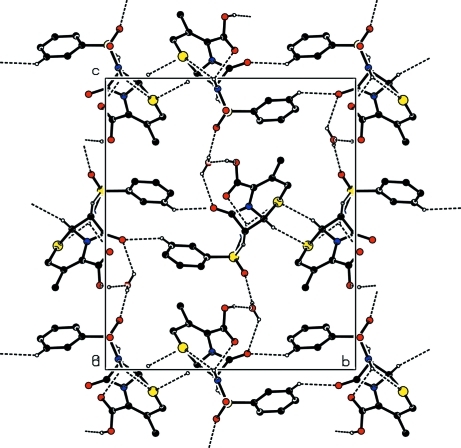
The packing and hydrogen bonding of the title compound viewed down a axis. Hydrogen atoms not involved in hydrogen bonding have been omitted for clarity.

## supplementary crystallographic information

### Comment

One of the building blocks of cephalosporin antibiotics is 7-amino-deacetoxycephalosporanic acid (7-ADCA). It is currently produced from penicillin G using an elaborate chemical ring-expansion step followed by an enzyme-catalyzed hydrolysis. However, 7-ADCA-like components can also be produced by direct fermentation (Schroen *et al.* 2000).

In the title molecule (I) shown in Fig. 1, the β-lactam unit (N1/C6–C8) has a twisted conformation with the dihedral angles of 164.7 (4)° and 164.7 (4)° between the planes N1 C6 C7 and C6 C8 C7 and the planes N1 C6 C8 and N1 C8 C7, respectively. The six-membered ring fused to the β-lactam unit, (N1/S1/C1/C2/C4/C6) is puckered with the puckering parameters (Cremer & Pople, 1975): Q_T_ = 0.618 (3) Å, θ = 54.2 (4) °, φ = 340.4 (5) °, respectively.

The crystal stucture is stabilized by intermolecular C—H···S, N—H···O, C—H···O and O—H···O hydrogen bonding interactions between symmetry-related molecules, forming a network in three dimensions (Table 1, Fig. 2). Furthermore, there is a weak C—H···π interaction [C3—H3B···*Cg*3(-*x* + 3/2, -*y* + 1, *z* + 1/2); H3B···*Cg*3 = 2.72 Å, C3···*Cg*3 = 3.640 (5) Å, C3—H3B···*Cg*3 = 161°, where *Cg*3 is a centroid of the phenyl ring C9–C14].

### Experimental

7-ADCA (1 g, 4.7 mmol) was dissolved in distilled water (20 ml) in a round bottom flask (50 ml). Na_2_CO_3_ (3*M*) solution was added to maintain the solution at pH 8–9, then benzene sulfonyl chloride (0.82 g, 4.7 mmol) was added dropwise to the solution and stirred at room temperature. As all benzene sulfonyl chloride was consumed, pH becomes stable at 8–9, which confirms the completion of reaction. Then pH was adjusted to 1–2, by using 3 N HCl. The precipitate obtained was filtered, washed with distilled water, dried and recrystalized in ethyl acetate. Light yellow prisms of (I) appeared after two days.

### Refinement

The H atoms of the water molecule were located in difference Fourier maps and were refined with O—H distances restrained to 0.83 (1) Å and H···H distances restrained to 1.30 (1) Å, with displacement parameters fixed at 1.5 times *U*_eq_ of the parent O atoms. H atom on O1 was calculated and refined with a riding model [O—H = 0.82Å and *U*_iso_(H) = 1.5*U*_eq_(O)]. The other H atoms were placed geometrically, with N—H = 0.86 Å, C—H = 0.96–0.98 Å, and included in the refinement using a riding model, with *U*_iso_(H) = 1.2 or 1.5*U*_eq_(parent atom).

### Crystal data

**Table:**

C_14_H_14_N_2_O_5_S_2_·H_2_O	*F*(000) = 776
*M**_r_* = 372.43	*D*_x_ = 1.426 Mg m^−^^3^
Orthorhombic, *P*2_1_2_1_2_1_	Mo *K*α radiation, λ = 0.71073 Å
Hall symbol: P 2ac 2ab	Cell parameters from 1402 reflections
*a* = 5.9535 (7) Å	θ = 2.8–17.3°
*b* = 15.8248 (19) Å	µ = 0.34 mm^−^^1^
*c* = 18.411 (2) Å	*T* = 296 K
*V* = 1734.6 (3) Å^3^	Prismatic, light yellow
*Z* = 4	0.20 × 0.20 × 0.20 mm

### Data collection

**Table:**

Bruker Kappa APEXII CCD area-detector diffractometer	1890 reflections with *I* > 2σ(*I*)
Radiation source: sealed tube	*R*_int_ = 0.064
graphite	θ_max_ = 26.8°, θ_min_ = 2.6°
φ and ω scans	*h* = −4→7
10847 measured reflections	*k* = −12→20
3572 independent reflections	*l* = −15→23

### Refinement

**Table:**

Refinement on *F*^2^	Hydrogen site location: inferred from neighbouring sites
Least-squares matrix: full	H atoms treated by a mixture of independent and constrained refinement
*R*[*F*^2^ > 2σ(*F*^2^)] = 0.055	*w* = 1/[σ^2^(*F*_o_^2^) + (0.0407*P*)^2^] where *P* = (*F*_o_^2^ + 2*F*_c_^2^)/3
*wR*(*F*^2^) = 0.109	(Δ/σ)_max_ < 0.001
*S* = 0.97	Δρ_max_ = 0.20 e Å^−^^3^
3572 reflections	Δρ_min_ = −0.25 e Å^−^^3^
226 parameters	Extinction correction: *SHELXL97* (Sheldrick, 2008), Fc^*^=kFc[1+0.001xFc^2^λ^3^/sin(2θ)]^-1/4^
3 restraints	Extinction coefficient: 0.0083 (10)
Primary atom site location: structure-invariant direct methods	Absolute structure: Flack (1983), 1425 Friedel pairs
Secondary atom site location: difference Fourier map	Flack parameter: −0.07 (11)

### Special details

**Table:**

Geometry. Bond distances, angles *etc*. have been calculated using the rounded fractional coordinates. All su's are estimated from the variances of the (full) variance-covariance matrix. The cell e.s.d.'s are taken into account in the estimation of distances, angles and torsion angles
Refinement. Refinement on *F*^2^ for ALL reflections except those flagged by the user for potential systematic errors. Weighted *R*-factors *wR* and all goodnesses of fit *S* are based on *F*^2^, conventional *R*-factors *R* are based on *F*, with *F* set to zero for negative *F*^2^. The observed criterion of *F*^2^ > σ(*F*^2^) is used only for calculating -*R*-factor-obs *etc*. and is not relevant to the choice of reflections for refinement. *R*-factors based on *F*^2^ are statistically about twice as large as those based on *F*, and *R*-factors based on ALL data will be even larger.

### Fractional atomic coordinates and isotropic or equivalent isotropic displacement parameters (Å^2^)

**Table:**

	*x*	*y*	*z*	*U*_iso_*/*U*_eq_	
S1	0.8308 (2)	0.30975 (7)	0.07531 (7)	0.0628 (5)	
S2	1.06325 (17)	0.48679 (7)	−0.11299 (6)	0.0452 (4)	
O1	0.1740 (7)	0.4774 (2)	0.21094 (17)	0.0880 (16)	
O2	0.1461 (5)	0.51706 (18)	0.09585 (15)	0.0573 (11)	
O3	0.6341 (5)	0.56743 (17)	0.05463 (18)	0.0719 (13)	
O4	1.3013 (4)	0.48188 (19)	−0.11039 (15)	0.0595 (11)	
O5	0.9420 (5)	0.44363 (17)	−0.16919 (15)	0.0554 (10)	
N1	0.5130 (5)	0.42642 (19)	0.06042 (18)	0.0410 (11)	
N2	0.9775 (5)	0.44881 (19)	−0.03638 (18)	0.0447 (12)	
C1	0.6086 (8)	0.2712 (3)	0.1326 (3)	0.072 (2)	
C2	0.4335 (7)	0.3333 (3)	0.1575 (2)	0.0500 (17)	
C3	0.2901 (9)	0.2995 (3)	0.2184 (2)	0.077 (2)	
C4	0.3988 (6)	0.4074 (3)	0.1247 (2)	0.0427 (16)	
C5	0.2262 (7)	0.4725 (3)	0.1418 (3)	0.0507 (17)	
C6	0.6407 (7)	0.3665 (2)	0.0176 (2)	0.0500 (17)	
C7	0.6267 (6)	0.4950 (3)	0.0341 (2)	0.0480 (16)	
C8	0.7372 (7)	0.4416 (3)	−0.0250 (2)	0.0470 (17)	
C9	0.9814 (6)	0.5938 (2)	−0.1135 (2)	0.0430 (14)	
C10	1.1108 (7)	0.6515 (3)	−0.0765 (2)	0.0587 (17)	
C11	1.0450 (10)	0.7349 (3)	−0.0734 (3)	0.074 (2)	
C12	0.8513 (10)	0.7598 (3)	−0.1058 (3)	0.069 (2)	
C13	0.7206 (8)	0.7021 (3)	−0.1421 (3)	0.0683 (19)	
C14	0.7853 (7)	0.6178 (3)	−0.1457 (3)	0.0577 (19)	
OW1	0.8355 (9)	0.5874 (3)	0.2110 (3)	0.128 (2)	
H1	0.07960	0.51450	0.21690	0.1320*	
H1A	0.53260	0.22610	0.10690	0.0860*	
H1B	0.67690	0.24650	0.17550	0.0860*	
H2	1.07130	0.43340	−0.00340	0.0530*	
H3A	0.17150	0.33880	0.22870	0.1160*	
H3B	0.38080	0.29200	0.26100	0.1160*	
H3C	0.22680	0.24620	0.20430	0.1160*	
H6	0.54600	0.32940	−0.01210	0.0600*	
H8	0.65710	0.44840	−0.07110	0.0560*	
H10	1.24240	0.63430	−0.05370	0.0710*	
H11	1.13350	0.77410	−0.04910	0.0890*	
H12	0.80690	0.81610	−0.10330	0.0830*	
H13	0.58820	0.71950	−0.16430	0.0820*	
H14	0.69620	0.57840	−0.16970	0.0690*	
HW1	0.706 (6)	0.587 (5)	0.228 (3)	0.1920*	
HW2	0.819 (12)	0.614 (4)	0.172 (2)	0.1920*	

### Atomic displacement parameters (Å^2^)

**Table:**

	*U*^11^	*U*^22^	*U*^33^	*U*^12^	*U*^13^	*U*^23^
S1	0.0590 (8)	0.0401 (6)	0.0892 (10)	0.0106 (6)	0.0078 (8)	0.0044 (6)
S2	0.0366 (6)	0.0512 (7)	0.0479 (7)	0.0031 (5)	0.0001 (6)	0.0012 (6)
O1	0.104 (3)	0.110 (3)	0.050 (2)	0.032 (3)	0.014 (2)	−0.012 (2)
O2	0.0538 (19)	0.0611 (19)	0.057 (2)	0.0140 (18)	0.0046 (17)	−0.0017 (17)
O3	0.062 (2)	0.0368 (17)	0.117 (3)	0.0035 (17)	0.029 (2)	−0.0053 (18)
O4	0.0328 (16)	0.080 (2)	0.0656 (19)	0.0071 (16)	0.0052 (15)	0.0055 (18)
O5	0.0591 (18)	0.0585 (17)	0.0485 (19)	−0.0021 (16)	−0.0076 (17)	−0.0051 (15)
N1	0.0398 (19)	0.0353 (18)	0.048 (2)	0.0017 (17)	0.0067 (18)	−0.0016 (17)
N2	0.029 (2)	0.054 (2)	0.051 (2)	0.0090 (16)	0.0011 (16)	0.0119 (18)
C1	0.077 (4)	0.048 (3)	0.091 (4)	−0.003 (3)	−0.002 (3)	0.012 (3)
C2	0.043 (3)	0.057 (3)	0.050 (3)	−0.010 (3)	0.000 (2)	0.007 (2)
C3	0.080 (4)	0.086 (4)	0.066 (3)	−0.013 (3)	−0.005 (3)	0.017 (3)
C4	0.038 (2)	0.050 (3)	0.040 (3)	−0.005 (2)	−0.001 (2)	−0.002 (2)
C5	0.040 (3)	0.063 (3)	0.049 (3)	−0.005 (2)	0.004 (2)	−0.012 (3)
C6	0.048 (3)	0.046 (3)	0.056 (3)	−0.010 (2)	0.000 (2)	−0.011 (2)
C7	0.041 (3)	0.039 (2)	0.064 (3)	0.005 (2)	0.003 (2)	0.010 (2)
C8	0.037 (3)	0.054 (3)	0.050 (3)	0.006 (2)	−0.003 (2)	0.004 (2)
C9	0.037 (2)	0.044 (2)	0.048 (3)	−0.005 (2)	0.004 (2)	0.007 (2)
C10	0.052 (3)	0.058 (3)	0.066 (3)	−0.005 (3)	−0.012 (3)	0.001 (3)
C11	0.094 (4)	0.046 (3)	0.083 (4)	−0.015 (3)	−0.020 (4)	−0.004 (3)
C12	0.082 (4)	0.044 (3)	0.081 (4)	0.001 (3)	0.009 (4)	0.000 (3)
C13	0.053 (3)	0.062 (3)	0.090 (4)	0.012 (3)	−0.012 (3)	0.013 (3)
C14	0.047 (3)	0.048 (3)	0.078 (4)	−0.004 (2)	−0.004 (3)	0.002 (2)
OW1	0.152 (4)	0.104 (3)	0.129 (4)	0.035 (3)	0.085 (4)	0.000 (3)

### Geometric parameters (Å, °)

**Table:**

S1—C1	1.799 (5)	C6—C8	1.535 (6)
S1—C6	1.793 (4)	C7—C8	1.527 (6)
S2—O4	1.420 (3)	C9—C14	1.363 (6)
S2—O5	1.435 (3)	C9—C10	1.375 (6)
S2—N2	1.616 (3)	C10—C11	1.378 (7)
S2—C9	1.762 (3)	C11—C12	1.357 (8)
O1—C5	1.313 (6)	C12—C13	1.373 (7)
O2—C5	1.200 (6)	C13—C14	1.390 (7)
O3—C7	1.208 (5)	C1—H1B	0.9700
O1—H1	0.8200	C1—H1A	0.9700
OW1—HW2	0.84 (5)	C3—H3C	0.9600
OW1—HW1	0.83 (4)	C3—H3A	0.9600
N1—C4	1.398 (5)	C3—H3B	0.9600
N1—C7	1.368 (5)	C6—H6	0.9800
N1—C6	1.449 (5)	C8—H8	0.9800
N2—C8	1.450 (5)	C10—H10	0.9300
N2—H2	0.8600	C11—H11	0.9300
C1—C2	1.504 (7)	C12—H12	0.9300
C2—C4	1.335 (6)	C13—H13	0.9300
C2—C3	1.507 (6)	C14—H14	0.9300
C4—C5	1.489 (6)		
			
S1···N2	3.136 (3)	C5···OW1^iii^	3.216 (7)
S1···C5^i^	3.698 (5)	C7···O4^iii^	3.297 (5)
S1···H2	2.8200	C7···O2^i^	3.313 (5)
S1···H6^ii^	2.8000	C7···O2	3.099 (5)
O1···OW1^iii^	2.663 (6)	C8···C14	3.577 (7)
O1···C3	2.902 (6)	C8···O4^iii^	3.100 (5)
OW1···O2^i^	3.025 (6)	C11···O3^vii^	3.192 (6)
OW1···O3	3.135 (6)	C12···O3^vii^	3.346 (6)
OW1···C5^i^	3.216 (7)	C14···C8	3.577 (7)
OW1···O5^iv^	2.799 (6)	C4···H2^iii^	3.0900
OW1···O1^i^	2.663 (6)	C5···H3A	2.6700
O2···N1	2.693 (4)	C5···H2^iii^	2.8900
O2···C7^iii^	3.313 (5)	C9···H3B^vi^	3.0500
O2···C7	3.099 (5)	C12···H3B^vi^	3.0400
O2···OW1^iii^	3.025 (6)	C13···H3B^vi^	2.9700
O2···O3	3.107 (4)	C14···H3B^vi^	2.9900
O2···N2^iii^	2.846 (4)	H1···HW2^iii^	2.3600
O3···O2	3.107 (4)	H1···OW1^iii^	1.8600
O3···OW1	3.135 (6)	H1···HW1^iii^	2.5100
O3···C5	3.276 (6)	HW1···O5^iv^	2.14 (5)
O3···C12^v^	3.346 (6)	HW1···H1^i^	2.5100
O3···N2	3.242 (4)	H1A···H3C	2.5800
O3···C11^v^	3.192 (6)	H1B···H3B	2.4700
O4···C7^i^	3.297 (5)	H2···O2^i^	2.3000
O4···C8^i^	3.100 (5)	H2···N1^i^	2.8800
O5···OW1^vi^	2.799 (6)	H2···S1	2.8200
O1···H3A	2.2200	H2···C5^i^	2.8900
OW1···H1^i^	1.8600	H2···C4^i^	3.0900
O2···HW2^iii^	2.85 (6)	HW2···O3	2.54 (5)
O2···H2^iii^	2.3000	HW2···O2^i^	2.85 (6)
O2···H12^v^	2.8100	HW2···H1^i^	2.3600
O3···HW2	2.54 (5)	H3A···O1	2.2200
O3···H12^v^	2.8300	H3A···C5	2.6700
O3···H11^v^	2.5100	H3B···C12^iv^	3.0400
O4···H10	2.6500	H3B···C14^iv^	2.9900
O4···H8^i^	2.3000	H3B···C9^iv^	3.0500
O5···HW1^vi^	2.14 (5)	H3B···C13^iv^	2.9700
O5···H14	2.5900	H3B···H1B	2.4700
O5···H8	2.4800	H3C···H1A	2.5800
N1···O2	2.693 (4)	H6···S1^viii^	2.8000
N2···O2^i^	2.846 (4)	H8···O5	2.4800
N2···S1	3.136 (3)	H8···O4^iii^	2.3000
N2···O3	3.242 (4)	H10···O4	2.6500
N1···H2^iii^	2.8800	H11···O3^vii^	2.5100
C3···O1	2.902 (6)	H12···O3^vii^	2.8300
C5···O3	3.276 (6)	H12···O2^vii^	2.8100
C5···S1^iii^	3.698 (5)	H14···O5	2.5900
			
C1—S1—C6	93.1 (2)	S2—C9—C14	120.5 (3)
O4—S2—O5	120.03 (18)	C10—C9—C14	120.7 (4)
O4—S2—N2	105.40 (17)	C9—C10—C11	119.8 (4)
O4—S2—C9	109.19 (18)	C10—C11—C12	120.1 (5)
O5—S2—N2	107.07 (17)	C11—C12—C13	120.2 (5)
O5—S2—C9	108.33 (17)	C12—C13—C14	120.3 (5)
N2—S2—C9	105.95 (17)	C9—C14—C13	118.9 (4)
C5—O1—H1	110.00	S1—C1—H1A	108.00
HW1—OW1—HW2	103 (6)	S1—C1—H1B	108.00
C6—N1—C7	93.8 (3)	C2—C1—H1B	108.00
C4—N1—C6	125.1 (3)	H1A—C1—H1B	107.00
C4—N1—C7	135.4 (3)	C2—C1—H1A	108.00
S2—N2—C8	117.8 (3)	C2—C3—H3B	109.00
C8—N2—H2	121.00	C2—C3—H3C	109.00
S2—N2—H2	121.00	H3A—C3—H3B	109.00
S1—C1—C2	117.8 (3)	H3A—C3—H3C	110.00
C1—C2—C4	122.9 (4)	H3B—C3—H3C	109.00
C3—C2—C4	124.1 (4)	C2—C3—H3A	109.00
C1—C2—C3	112.8 (4)	S1—C6—H6	113.00
N1—C4—C2	119.8 (4)	C8—C6—H6	113.00
N1—C4—C5	111.5 (4)	N1—C6—H6	113.00
C2—C4—C5	128.2 (4)	C6—C8—H8	110.00
O1—C5—C4	114.2 (4)	C7—C8—H8	110.00
O2—C5—C4	122.1 (5)	N2—C8—H8	110.00
O1—C5—O2	123.7 (4)	C11—C10—H10	120.00
S1—C6—N1	109.7 (3)	C9—C10—H10	120.00
S1—C6—C8	117.0 (3)	C10—C11—H11	120.00
N1—C6—C8	88.2 (3)	C12—C11—H11	120.00
N1—C7—C8	91.5 (3)	C13—C12—H12	120.00
O3—C7—N1	131.3 (4)	C11—C12—H12	120.00
O3—C7—C8	137.1 (4)	C12—C13—H13	120.00
N2—C8—C7	119.0 (3)	C14—C13—H13	120.00
C6—C8—C7	84.4 (3)	C9—C14—H14	121.00
N2—C8—C6	120.3 (4)	C13—C14—H14	121.00
S2—C9—C10	118.7 (3)		
			
C6—S1—C1—C2	−46.4 (4)	S1—C1—C2—C3	−166.4 (3)
C1—S1—C6—N1	57.8 (3)	S1—C1—C2—C4	18.8 (6)
C1—S1—C6—C8	156.1 (3)	C1—C2—C4—N1	6.2 (6)
O4—S2—N2—C8	−176.8 (3)	C1—C2—C4—C5	177.2 (4)
O5—S2—N2—C8	−47.9 (3)	C3—C2—C4—N1	−168.1 (4)
C9—S2—N2—C8	67.6 (3)	C3—C2—C4—C5	3.0 (7)
O4—S2—C9—C10	−32.7 (4)	N1—C4—C5—O1	−155.7 (4)
O4—S2—C9—C14	151.9 (4)	N1—C4—C5—O2	23.2 (6)
O5—S2—C9—C10	−165.0 (3)	C2—C4—C5—O1	32.6 (6)
O5—S2—C9—C14	19.5 (4)	C2—C4—C5—O2	−148.5 (5)
N2—S2—C9—C10	80.4 (3)	S1—C6—C8—N2	19.3 (5)
N2—S2—C9—C14	−95.1 (4)	S1—C6—C8—C7	−101.1 (3)
C6—N1—C4—C2	11.5 (6)	N1—C6—C8—N2	130.5 (4)
C6—N1—C4—C5	−161.0 (4)	N1—C6—C8—C7	10.1 (3)
C7—N1—C4—C2	−134.5 (5)	O3—C7—C8—N2	45.1 (7)
C7—N1—C4—C5	53.1 (6)	O3—C7—C8—C6	166.7 (5)
C4—N1—C6—S1	−49.9 (4)	N1—C7—C8—N2	−132.3 (4)
C4—N1—C6—C8	−168.0 (3)	N1—C7—C8—C6	−10.7 (3)
C7—N1—C6—S1	106.9 (3)	S2—C9—C10—C11	−177.2 (4)
C7—N1—C6—C8	−11.2 (3)	C14—C9—C10—C11	−1.7 (6)
C4—N1—C7—O3	−13.7 (7)	S2—C9—C14—C13	177.0 (4)
C4—N1—C7—C8	164.0 (4)	C10—C9—C14—C13	1.6 (7)
C6—N1—C7—O3	−166.4 (4)	C9—C10—C11—C12	1.0 (7)
C6—N1—C7—C8	11.3 (3)	C10—C11—C12—C13	−0.3 (8)
S2—N2—C8—C6	143.8 (3)	C11—C12—C13—C14	0.2 (8)
S2—N2—C8—C7	−114.9 (3)	C12—C13—C14—C9	−0.8 (8)

Symmetry codes: (i) *x*+1, *y*, *z*; (ii) *x*+1/2, −*y*+1/2, −*z*; (iii) *x*−1, *y*, *z*; (iv) −*x*+3/2, −*y*+1, *z*+1/2; (v) *x*−1/2, −*y*+3/2, −*z*; (vi) −*x*+3/2, −*y*+1, *z*−1/2; (vii) *x*+1/2, −*y*+3/2, −*z*; (viii) *x*−1/2, −*y*+1/2, −*z*.

### Hydrogen-bond geometry (Å, °)

**Table:**

*D*—H···*A*	*D*—H	H···*A*	*D*···*A*	*D*—H···*A*
O1—H1···OW1^iii^	0.82	1.86	2.663 (6)	166
OW1—HW1···O5^iv^	0.83 (4)	2.14 (5)	2.799 (6)	136 (5)
N2—H2···S1	0.86	2.82	3.136 (3)	103
N2—H2···O2^i^	0.86	2.30	2.846 (4)	122
OW1—HW2···O3	0.84 (5)	2.54 (5)	3.135 (6)	129 (5)
C3—H3A···O1	0.96	2.22	2.902 (6)	127
C6—H6···S1^viii^	0.98	2.80	3.756 (4)	165
C8—H8···O4^iii^	0.98	2.30	3.100 (5)	138
C8—H8···O5	0.98	2.48	2.922 (5)	107
C11—H11···O3^vii^	0.93	2.51	3.192 (6)	130
C14—H14···O5	0.93	2.59	2.942 (5)	103
C3—H3B···Cg3^iv^	0.96	2.72	3.640 (5)	161

Symmetry codes: (iii) *x*−1, *y*, *z*; (iv) −*x*+3/2, −*y*+1, *z*+1/2; (i) *x*+1, *y*, *z*; (viii) *x*−1/2, −*y*+1/2, −*z*; (vii) *x*+1/2, −*y*+3/2, −*z*.
